# *CHRNA3* rs6495308 Genotype as an Effect Modifier of the Association between Daily Cigarette Consumption and Hypertension in Chinese Male Smokers

**DOI:** 10.3390/ijerph120404156

**Published:** 2015-04-14

**Authors:** Xiao-Ying Wu, Shan-Yu Zhou, Zhong-Zheng Niu, Tao Liu, Chuan-Bo Xie, Wei-Qing Chen

**Affiliations:** 1 Department of Biostatistics and Epidemiology, School of Public Health, Sun Yat-Sen University, Guangzhou, Guangdong 510080, China; E-Mails: xiaoyingwu2014@hotmail.com (X.Y.W.); niuxshijia@hotmail.com (Z.Z.N.); chenwq@mail.sysu.edu.cn (W.Q.C.); 2 Guangdong Prevention and Treatment Center for Occupational Diseases, Guangzhou, Guangdong 510000, China; E-Mail: sandyzhou168@sina.com; 3 Guangdong Provincial Institute of Public Health, Guangdong Provincial Center for Disease Control and Prevention, Guangzhou, Guangdong 510000, China; E-Mail: gztt_2002@163.com; 4 Division of Behavioral Medicine, School of Medicine and Biomedical Sciences, State University of New York at Buffalo, Buffalo, New York, NY 14228, USA; E-Mail: xieechuanbo@126.com

**Keywords:** cigarette smoking, nicotine acetylcholine receptor, genotype, hypertension

## Abstract

Cigarette smoking is an important risk factor for hypertension. However, the effects on hypertension of the interaction between smoking and the genotype of the nicotinic acetylcholine receptor gene are unclear. The purpose of this study is to determine whether the *CHRNA3* rs6495308 genotype affects the association between daily cigarette consumption and hypertension. We recruited 947 male smokers in southern China and used a questionnaire administered in face to face interviews to obtain information on their socio-demographic characteristics and smoking behavior. Blood samples were collected to test for *CHRNA3* rs6495308 genotype variations. Three blood-pressure measurements were taken for each participant, and the average values recorded. We found that, compared with light smoking (<15 cigarettes per day), heavy smoking (≥15 cigarettes per day) yielded a greater risk of hypertension. We also observed that the interaction between daily cigarette consumption and the *CHRNA3* rs6495308 genotype may affect hypertension. Heavy smokers with the homozygous mutant *CHRNA3* rs6495308 genotype exhibited a significantly greater risk of hypertension than light smokers with wild-type *CHRNA3* rs6495308 genotypes. The positive interaction between heavy smoking and the homozygous mutant *CHRNA3* rs6495308 genotype was found to affect the likelihood of hypertension in Chinese male smokers.

## 1. Introduction

Hypertension, generally defined as high blood pressure, is a multifactorial disease induced by the interaction of genetic and environmental risk factors [1]. Previous studies have shown that cigarette smoking is associated with an increased risk of hypertension [2,3,4,5]. 

Cigarette smoking is a genetically influenced addictive behavior [6], and nicotine is the main component of cigarette smoke responsible for smoking dependence [7]. Therefore, research on the moderating effects of nicotine-related genetic factors on the association between cigarette smoking and hypertension has important implications for public health. The nicotine-related genetic factors *CYP2A6* and *CHRNA5-CHRNA3-CHRNB4* are significantly related to smoking behavior. The results of several studies indicate that *CYP2A6* polymorphism and polymorphisms in the enzymes that encode the *CYP2A6* gene and metabolize nicotine (cytochrome P450 2A6) [8,9,10] are associated with the number of cigarettes smoked per day [10,11], and moderate the relationship between cigarette smoking and smoking-related cancers such as lung cancer [11,12]. In addition, genetic variation in the nicotinic acetylcholine receptor (nAChR) gene cluster *CHRNA5-CHRNA3-CHRNB4* has been shown to be significantly associated with nicotine dependence [13,14], smoking quantity [15,16,17,18,19,20,21] and smoking-related diseases such as chronic obstructive pulmonary disease [21], peripheral arterial disease [22] and lung cancer [22,23,24,25,26]. For example, the rs6495308 single-nucleotide polymorphism (SNP) in the *CHRNA3* gene has been found to influence smoking quantity in the Korean population [18].

In previous studies of Chinese smokers, we have shown that the interaction between individuals’ *CYP2A6* genotype and smoking quantity has significant effects on type-2 diabetes [27], abdominal obesity [28] and hypertension [29]. However, little work has been done to determine whether the interaction between the *CHRNA5-CHRNA3-CHRNB4* genotype and smoking quantity affects the risk of hypertension in Chinese smokers. To fill this gap in the research, we focus in the current study on the effects on hypertension of the relationship between rs6495308 genotypes of the *CHRNA3* gene and daily cigarette consumption. The results may extend our knowledge of the relationship between smoking and hypertension.

## 2. Methods

### 2.1. Participants

The participants were recruited from a community-based chronic disease study conducted in Guangzhou and Zhuhai in southern China from July 2006 to June 2007 [30]. A four-stage sampling method was used to select a representative sample of the community resident in these two cities. In stage one, six administrative regions from Guangzhou and one from Zhuhai were randomly sampled. In stage two, one street district was randomly selected from each of the seven sampled administrative regions. In stage three, two communities were randomly sampled from each selected street district. In stage four, residents aged ≥20 years living in Guangzhou or Zhuhai for ≥5 years were invited to participate in the survey. A total of 7293 adult residents (2465 males and 4828 females) were sampled. The lower proportion of males in the enrollees might be owed that, compared with females, males were busier with their work and less paid attention to their health, which resulted in their lower participation in the survey. 

**Figure 1:**
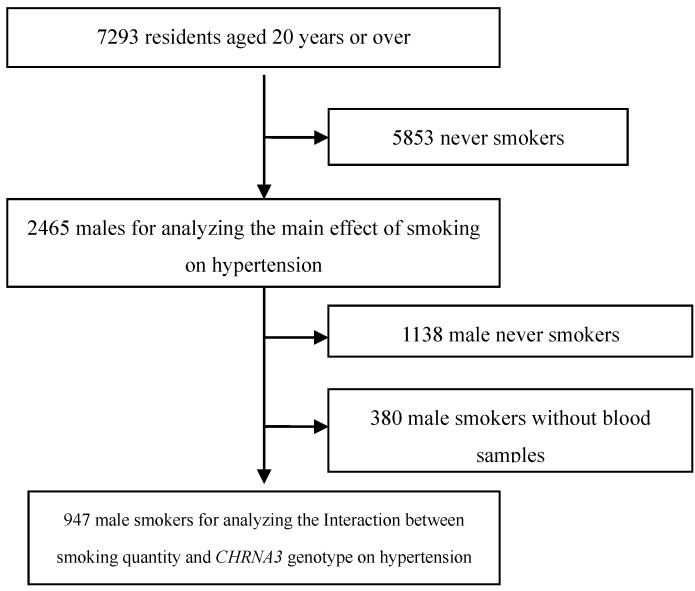
Participant selection.

To minimize the confounding effects of smoking status and gender, only the data provided by male smokers were used to assess the interaction effects of daily cigarette consumption and *CHRNA5-CHRNA3-CHRNB4* polymorphism on hypertension. Among the 2465 males, 1327 were smokers, of them, 380 failed to provide the blood samples required to detect *CHRNA5-CHRNA3-CHRNB4* polymorphisms, and were thus excluded from the study. The final sample comprised 947 male smokers (Figure 1). The study was conducted in accordance with the Declaration of Helsinki, and the protocol was approved by the Ethics Committee of Sun Yat-Sen University in Guangzhou, China (Approval No. 2013 [8]). The written informed consent was obtained from all of the participants before the survey was started.

### 2.2. Data Collection

Structured questionnaires were administered in interviews by well-trained clinicians to obtain information on the participants’ socio-demographic characteristics (e.g., age, education level, occupation and family income), family history of hypertension, smoking behavior and lifestyle. Anthropometric indices were measured and blood specimens were collected for genotyping. Three blood-pressure measurements were taken for each participant, and the average values recorded. The participants were requested not to smoke until the blood-pressure measurements were complete [31].

### 2.3. Definitions Relating to Smoking Behavior

A smoker is defined as an individual who has smoked more than 100 cigarettes in his/her lifetime [32]. We measured daily cigarette consumption by the average number of cigarettes smoked per day. The median number of cigarettes smoked per day (15) was used to divide the smokers into two groups: light smokers (who smoked <15 cigarettes per day) and heavy smokers (who smoked ≥ 15 cigarettes per day). 

### 2.4. Definition of Hypertension

According to the 2007 Practice Guidelines released by the European Society of Hypertension and the European Society of Cardiology [33], hypertension is defined as systolic blood pressure of at least 140 mmHg and/or diastolic blood pressure of at least 90 mmHg, or self-reported diagnosed hypertension requiring antihypertensive drugs. We excluded individuals with secondary hypertension, coronary heart disease and/or diabetes from our study.

### 2.5. Genotyping 

The rs6495308 SNP in the *CHRNA3* gene was selected for analysis after applying the following criteria: (a) reported in more than one study to have a functional effect on or association with smoking addition or nicotine dependence [34,35,36]; (b) minor allele frequency greater than 5% in the Han Chinese population of Beijing, according to the HapMap database; and (c) an R^2^ value smaller than 0.85, with other markers. 

Each participant provided a 10 mL venous blood sample, from which we extracted genomic DNA using the Universal Genomic DNA Extraction Kit Ver. 3.0 (Takara, Dalian, China). The genotyping was performed by improved multiple ligase detection reaction (iMLDR). The ABI Polymerase Chain Reaction (PCR) System 9600 (Applied Biosystems, Foster City, CA, USA) was used to conduct multiple PCR with 10-μL samples, each containing 1 μL of genomic DNA, 1 μL of PCR primer, 1 × GC-I buffer (Takara), 3.0 mM of Mg^2+^, 0.3 mM of dNTP and 1 U of HotStarTaq polymerase (Qiagen, Chatsworth, CA, USA). The cycling parameters were as follows: 95 ºC for 2 min; 11 cycles × (94 ºC for 20 s, 65 ºC–0.5 ºC in a 40-s cycle, 72 ºC for 1 min 30 s); 24 cycles × (94 ºC for 20 s, 59 ºC for 30 s, 72 ºC for 1 min 30 s); and a final extension step at 72 °C for 2 min. Next, we added 1 U of shrimp alkaline phosphatase enzyme and 1 U of exonuclease-I enzyme to the products of the multiple PCR, and placed them in a water bath at 37 ºC for 1 hour and 75 ºC for 15 min for enzyme inactivation. For each PCR product, the iMLDR was performed in a final volume of 10 μL, comprising 2 μL of PCR product, 1 μL of 10 × buffer, 0.25 ul of Taq DNA ligase (New England Biolabs, Beverly, MA., USA), 0.4 μL of 5’ probe mixture (1 µM), 0.4 μL of 3’ probe mixture (2 µM) and 6 μL of ddH_2_O. The iMLDR parameters were as follows: 38 cycles at 94ºC for 1 min and 56ºC for 4 min. The products were submitted for sequencing with an ABI3730XL sequencer, and the results were analyzed using the software program GeneMapper 4.0 (Applied Biosystems, Foster, CA, USA).

### 2.6. Statistical Analysis

The categorical variables were evaluated as percentages of participants with the respective attributes. Chi-square tests were performed to test the differences between the characteristics of the cases and the controls. 

A series of binary logistic regression models were implemented with a sample of 947 smokers to assess the effects on hypertension (0 = no, 1 = yes) of the interaction between daily cigarette consumption (divided into two groups: light smokers, who consumed <15 cigarettes per day, and heavy smokers, who consumed ≥15 cigarettes per day) and *CHRNA3* rs6495308 genotype (homozygous wild-type, CC; heterozygous mutant, CT; and homozygous mutant, TT). After controlling for potential confounding effects, we added daily cigarette consumption and *CHRNA3* rs6495308 genotype to the model to test their main effects, followed by an interaction term between smoking quantity and the *CHRNA5* rs684513 genotype.

Next, the smokers were further divided into six groups according to their daily cigarette consumption and *CHRNA3* rs6495308 genotype. Light smokers with the homozygous wild-type *CHRNA3* rs6495308 genotype were taken as the reference group, and the sizes of the effects on hypertension of the other five groups were evaluated by binary logistic regression. We controlled for potential confounding effects. A three-dimensional bar graph of the resulting six odds ratios (ORs) was used to present the separate effects of exposure. The potential confounding factors were age, occupation, education level, a family history of hypertension, family income, physical exercise, alcohol consumption, age at smoking initiation and body-mass index (BMI).

The frequencies of the *CHRNA3* rs6495308 genotypes were in the Hardy-Weinberg equilibrium (*p* > 0.05). All of the *p* values were two-sided, and the significance level was set at *p* = 0.05. Version 17.0 of the Statistical Package for the Social Sciences (SPSS, Inc., Chicago, IL, USA) was used for the whole process of analysis.

## 3. Results

### 3.1. Characteristics of Cases and Controls

Significant differences in age, occupation, physical exercise and BMI were found between the cases and the controls (Table 1).

### 3.2. Association between Smoking Quantity and Hypertension

As shown in Table 2, heavy smokers were found to face a significantly higher risk of hypertension than light smokers (OR = 1.39, 95% CI = 1.06–1.83) after adjusting for a family history of hypertension, physical exercise, family income and age at smoking initiation.

**Table 1:** Comparison of the characteristics of the cases and controls.

Variables	Controls (N = 597)		Cases (N = 350)		*χ^2^*	*p*
	N	%	N	%		
Age (years)						
<30	17	2.85	2	0.57	67.939	<0.001
30–39	78	13.07	17	4.86		
40–49	127	21.27	42	12		
50–59	202	33.84	110	31.43		
60–69	143	24.95	127	36.29		
≥70	30	5.03	52	14.86		
Occupation						
Office worker	38	6.37	19	5.43	21.526	0.001
Farmer	61	10.22	31	8.86		
Technician	24	4.02	20	5.71		
Service provider	84	14.07	31	8.86		
Production worker	154	25.8	74	21.14		
Retired	144	24.12	128	36.57		
Unemployed	92	15.41	47	13.43		
Education level						
Illiterate	10	1.68	5	1.43	6.604	0.158
Elementary school	86	14.41	68	19.43		
Junior middle school	202	33.84	122	34.86		
Senior middle school or vocational secondary school	197	33	111	31.71		
College or above	102	17.09	44	12.57		
Family history of hypertension						
No	403	67.5	219	62.57	2.382	0.123
Yes	194	32.5	131	37.43		
Family income (monthly per person, RMB)						
<1000	79	14.74	45	14.11	6.212	0.102
1000 ~ 4999	339	63.25	224	70.22		
5000 ~ 8999	98	18.28	44	13.79		
≥9000	20	3.73	6	1.88		
Physical exercise						
No	222	37.19	100	28.57	7.297	0.007
Yes	375	62.81	250	71.43		
Alcohol consumption						
No	384	64.32	227	64.86	5.54	0.063
Yes	180	30.15	91	26		
Former drinker	33	5.53	32	9.14		
BMI						
Normal	308	51.59	116	33.14	35.115	<0.001
Overweight	283	47.4	221	63.14		
Obese	6	1.01	13	3.71		
Age at smoking initiation (years)						
<20	229	38.36	115	32.86	2.887	0.089
≥20	368	61.64	235	67.14		
*CHRNA3* (rs6495308)						
CC	304	50.92	180	51.43	1.442	0.486
CT	244	40.87	134	38.29		
TT	49	8.21	36	10.29		

### 3.3. Association between CHRNA3 rs6495308 Genotype and Hypertension 

After adjusting for a family history of hypertension, physical exercise, family income and age at smoking initiation, the OR for hypertension of the smokers with the homozygous mutant *CHRNA3* rs6495308 genotype was 1.26 (95% CI = 0.79–2.02), higher than that of the smokers with the homozygous wild-type genotype. However, this difference was not statistically significant. 

### 3.4. Relationship Between Daily Cigarette Consumption and Hypertension Moderated by the CHRNA3 rs6495308 Genotype in Chinese Male Smokers

After adjusting for age, education level, occupation, a family history of hypertension, physical exercise, age at smoking initiation and BMI, we found the interaction term between daily cigarette consumption and *CHRNA3* rs6495308 genotype to be statistically significant (*p* < 0.05) (Table 2). 

**Figure 2:**
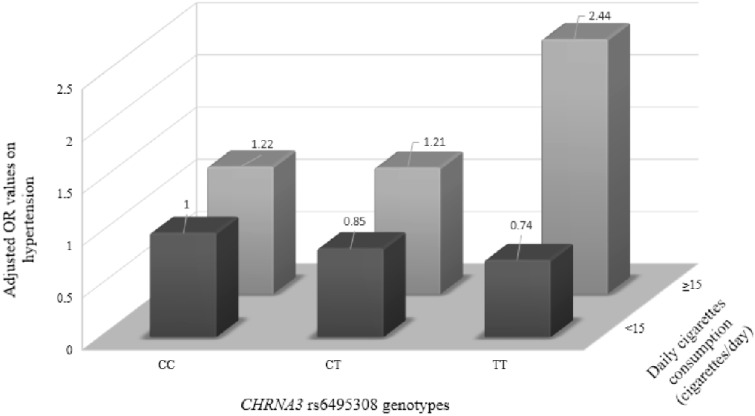
Cumulative interaction effect size of smoking quality and the *CHRNA3* rs6495308 genotype on hypertension in 947 Chinese male smokers. Six groups stratified by both daily cigarette consumption and *CHRNA3* rs6495308 genotype. Light smokers (<15 cigarettes per day) with the homozygous wild-type CC *CHRNA3* rs6495308 genotype make up the reference group (OR = 1). Adjusted variables: marital status, family income, family history of hypertension, physical exercise and age at smoking initiation. *p* = 0.036.

Smoking quantity and the *CHRNA3* rs6495308 genotype were used to divide the smokers into six groups. Significant differences were found between these groups (*p* = 0.036). After adjusting for marital status, family income, family history of hypertension, physical exercise and age at smoking initiation, heavy smokers with the homozygous mutant TT genotype of *CHRNA3* rs6495308 were found to have the highest risk of hypertension (OR = 2.44, 95% CI = 1.27–4.68) (Table 2 and Figure 2). 

## 4. Discussion

We identified significantly higher rates of hypertension among heavy smokers than light smokers. The distribution of *CHRNA3* rs6495308 did not differ significantly between the cases and the controls. However, the interaction between daily cigarette consumption and the *CHRNA3* genotype may affect hypertension, as heavy smokers with the homozygous mutant genotype of *CHRNA3* rs6495308 exhibited the highest risk of hypertension relative to the reference group.

### 4.1. Association between Smoking and Hypertension

A consensus has been achieved in previous studies on the effects of cigarette smoking on hypertension. According to two prospective studies, heavy smoking (≥15 cigarettes consumed per day) significantly increases the risk of hypertension [4,5]. In addition, the authors of an 11-year follow-up study conducted in Finland identified a dose-response relationship between smoking quantity and hypertension after controlling for waist circumference and alcohol use [3]. The results of a cross-sectional study with a Vietnamese population indicate that smokers who have smoked for more than 30 years, or have a lifetime smoking exposure of more than 20 pack years, face a significantly higher rate of hypertension than never-smokers, after controlling for age and BMI [37]. Consistent with these findings, the results of the current study indicate that heavy smoking is positively related to hypertension. Collectively, these studies demonstrate that smoking is an important risk factor in the development of hypertension.

**Table 2:** Effects of the interaction between smoking quantity and *CHRNA3* rs6495308 polymorphism on hypertension.

Indepenent Variables	Controls		Cases		Model 1	Model 2	Model 3
	N	%	N	%	Adjusted OR (95% CI)	Adjusted OR (95% CI)	*p*
**Hypertension ^#^**							
**Daily cigarette consumption**							
Light smoker (<15 cigarettes per day)	291	48.74	147	48.74	1		0.70
Heavy smoker (≥15 cigarettes per day)	306	51.25	203	51.25	1.39 (1.06–1.83) *		
***CHRNA3* (rs6495308)**							
CC	304	50.92	180	51.43	1	0.37
CT	244	40.87	134	38.29			0.93 (0.70–1.23)
TT	49	8.21	36	10.29			1.26 (0.79–2.02)
**Interaction term**							
Smoking quantity * *CHRNA3 rs649530* genotype							<**0.05**

**^#^** Dependent variable: hypertension (0 = no, 1 = yes); models 1 and 2: binary logistic regression models adjusted for a family history of hypertension, physical exercise, family income and age at smoking initiation; model 3: binary logistic regression model adjusted for age, education level, occupation, a family history of hypertension, physical exercise, age at smoking initiation and BMI; * *p* < 0.05.

### 4.2. Association between CHRNA5-CHRNA3-CHRNB4 Cluster Genotype and Hypertension

Few studies have been conducted on the effects of the nAChR genotype on hypertension and other cardiovascular diseases, and their results are inconsistent. Some researchers have found variations in the CHRNA5-CHRNA3-CHRNB4 gene cluster to have statistically significant effects on blood pressure and subclinical atherosclerosis [38,39]. However, the results of a cohort study indicate that the CHRNA5 rs1051730 polymorphism is not significantly linked with ischemic heart disease or ischemic stroke (RR = 0.9, 95% CI = 0.7–1.0; RR = 1.1, 95% CI = 0.8–1.4, respectively) [40]. Similarly, the findings of the current study suggest that no significant association exists between the CHRNA3 rs6495308 genotype and hypertension among Chinese smokers. However, the inconsistencies between these studies may be due to differences in the disease or ethnic population investigated. Therefore, further studies with larger samples and a broader population range are necessary to determine the true association between CHRNA5-CHRNA3-CHRNB4 polymorphism and cardiovascular disease.

### 4.3. Relationship between Daily Cigarette Consumption and Hypertension Moderated by the CHRNA5-CHRNA3-CHRNB4 Cluster Genotype

Numerous studies have been conducted on the effects of the association between smoking and nAChR-gene polymorphism on smoking-related diseases. The authors of a genome-wide association study of individuals of European descent found that a common SNP in the *CHRNA3* gene increases the risk of lung cancer by moderating smoking quantity and nicotine dependence. Each copy of the variant was found to increase smoking quantity by 0.095 units (*p* = 6.0 × 10^−2^), indirectly increasing the OR for lung cancer to 1.31 (*p* = 6.6 × 10^−8^) [22]. In another study, the interaction between genetic variation on 15q25 in the nAChR gene and smoking status was found to have significant effects on systolic blood pressure [41]. The results of the current study show that the interaction between daily cigarette consumption and the *CHRNA3* rs6495308 genotype significantly affects hypertension. The findings highlight the role of nicotine-related genetic factors in the pathology of smoking-related diseases.

The genetic contributors to some complex diseases are entirely dependent on environmental exposure, and may thus have substantial effects when specific environmental factors are present [42]. In this study, we focused on nicotine, the cigarette-combustion product primarily responsible for smoking dependence. However, further exploration of the components of cigarette smoke may reveal certain interaction effects. Polymorphism in the nAChR α3 gene (*CHRNA3*) is associated with a decrease in the expression of nAChRs in sympathetic nervous system cells [41], requiring smokers to smoke more cigarettes to achieve the same pleasant effects. As a result, smokers inhale more and more harmful cigarette-combustion products, such as oxidant chemicals, carbon monoxide, nitrogen oxides and heavy metals [7]. These harmful substances may cause a series of vascular reactions linked with the development of hypertension in smokers, such as oxidative stress, vasopressor effects, endothelial dysfunction, vascular injury, increased arterial stiffness and inflammation [43,44,45,46,47,48]. It should also be noted that in a recent study conducted with the same population, we found the association between smoking quantity and hypertension to be mediated by C-reactive protein, an inflammatory marker [49]. In addition, variation in the *CHRNA3* genotype may increase individuals’ susceptibility to the harmful constituents of tobacco smoke.

### 4.4. Limitations

The study has several limitations that may influence the interpretation of the results. First, its cross-sectional design prevents us from making inferences about causality. Second, we were unable to avoid information bias, as the data were collected retrospectively using a self-administrated questionnaire. Third, our exclusion of female smokers due to their very small presence in the sample may limit the generalizability of the results. However, we do not believe that this selection bias affected our findings. Fourth, we investigated just one SNP in the *CHRNA5-CHRNA3-CHRNB4* gene cluster, which may amplify its contribution and under-represent the joint effects of multiple SNPs in the cluster. Finally, we did not collect data on smokers who have given up smoking. Such individuals may have changed their lifestyles after being diagnosed with hypertension, and their use of antihypertensive medication may affect the measurement of blood pressure and the relationships between smoking quantity, *CHRNA3* genotype variation and hypertension. 

## 5. Conclusions

In sum, the findings of our study indicate that the *CHRNA3* rs6495308 genotype is an effect modifier of the association between daily cigarette consumption and hypertension in Chinese male smokers. This finding offers new insights into the causal relationship between smoking and hypertension.
